# Incorporation of Personal Single Nucleotide Polymorphism (SNP) Data into a National Level Electronic Health Record for Disease Risk Assessment, Part 3: An Evaluation of SNP Incorporated National Health Information System of Turkey for Prostate Cancer

**DOI:** 10.2196/medinform.3560

**Published:** 2014-08-19

**Authors:** Timur Beyan, Yeşim Aydın Son

**Affiliations:** ^1^Informatics InstituteDepartment of Health InformaticsMiddle East Technical UniversityAnkaraTurkey

**Keywords:** health information systems, clinical decision support systems, disease risk model, electronic health record, epigenetics, personalized medicine, single nucleotide polymorphism

## Abstract

**Background:**

A personalized medicine approach provides opportunities for predictive and preventive medicine. Using genomic, clinical, environmental, and behavioral data, the tracking and management of individual wellness is possible. A prolific way to carry this personalized approach into routine practices can be accomplished by integrating clinical interpretations of genomic variations into electronic medical records (EMRs)/electronic health records (EHRs). Today, various central EHR infrastructures have been constituted in many countries of the world, including Turkey.

**Objective:**

As an initial attempt to develop a sophisticated infrastructure, we have concentrated on incorporating the personal single nucleotide polymorphism (SNP) data into the National Health Information System of Turkey (NHIS-T) for disease risk assessment, and evaluated the performance of various predictive models for prostate cancer cases. We present our work as a three part miniseries: (1) an overview of requirements, (2) the incorporation of SNP data into the NHIS-T, and (3) an evaluation of SNP data incorporated into the NHIS-T for prostate cancer.

**Methods:**

In the third article of this miniseries, we have evaluated the proposed complementary capabilities (ie, knowledge base and end-user application) with real data. Before the evaluation phase, clinicogenomic associations about increased prostate cancer risk were extracted from knowledge sources, and published predictive genomic models assessing individual prostate cancer risk were collected. To evaluate complementary capabilities, we also gathered personal SNP data of four prostate cancer cases and fifteen controls. Using these data files, we compared various independent and model-based, prostate cancer risk assessment approaches.

**Results:**

Through the extraction and selection processes of SNP-prostate cancer risk associations, we collected 209 independent associations for increased risk of prostate cancer from the studied knowledge sources. Also, we gathered six cumulative models and two probabilistic models. Cumulative models and assessment of independent associations did not have impressive results. There was one of the probabilistic, model-based interpretation that was successful compared to the others. In envirobehavioral and clinical evaluations, we found that some of the comorbidities, especially, would be useful to evaluate disease risk. Even though we had a very limited dataset, a comparison of performances of different disease models and their implementation with real data as use case scenarios helped us to gain deeper insight into the proposed architecture.

**Conclusions:**

In order to benefit from genomic variation data, existing EHR/EMR systems must be constructed with the capability of tracking and monitoring all aspects of personal health status (genomic, clinical, environmental, etc) in 24/7 situations, and also with the capability of suggesting evidence-based recommendations. A national-level, accredited knowledge base is a top requirement for improved end-user systems interpreting these parameters. Finally, categorization using similar, individual characteristics (SNP patterns, exposure history, etc) may be an effective way to predict disease risks, but this approach needs to be concretized and supported with new studies.

## Introduction

In this miniseries, we share our work that aims to incorporate the personal single nucleotide polymorphism (SNP) data into a national level electronic health record, for example, the National Health Information System of Turkey (NHIS-T) for disease risk assessment based on genotyping information of patients.

First the literature review for SNP data incorporated electronic medical record (EMR)/electronic health record (EHR)s is presented. In addition, the requirements for the EMR/EHR systems in terms of the standardizations of terminologies and messaging are reviewed [1]. The need for a structured knowledge base, decision support approaches, systems for reporting, and risk assessment are addressed as well. Next, the NHIS-T system is overviewed, and architectural extensions to the NHIS-T for the integration of the SNP data are proposed [2]. Additionally, we have presented our design and developmental process for the complementary components of this system, for example, a knowledge base, Clinicogenomic Knowledge Base, (ClinGenKB), and end-user application, Clinicogenomic Web Application, (ClinGenWeb).

In this part, we evaluated these complementary components for prostate cancer using real, direct-to-consumer (DTC) SNP data files. We have first of all extracted and transformed clinicogenomic associations into knowledge base content, and determined assessment and reporting approaches to discern the disease risk at a personal level. Also an overall discussion of the results, limitations, and possibilities of our work covered in this miniseries is presented.

## Methods

### General Approach

In this article, we have focused on the evaluation of the developed ClinGenKB and ClinGenWeb for prostate cancer risk assessment.

Prostate cancer is the most common malignancy affecting men in the Western countries, it is highly heterogeneous and a multifactorial polygenic disease. The heterogeneous characteristics of prostate cancer could be partially explained by genetic factors [3]. In addition to genetic factors, age, race, family health history, endogenous hormones, diseases, environmental exposures, and various behavioral features are proposed in the literature as confounders of prostate cancer [4,5]. This complicated nature of prostate cancer, and burden on public health services, make it an ideal case to research the benefits of incorporating SNP data into an EHR for predictive, preventive, and personalized medicine approaches.

**Figure 1:**
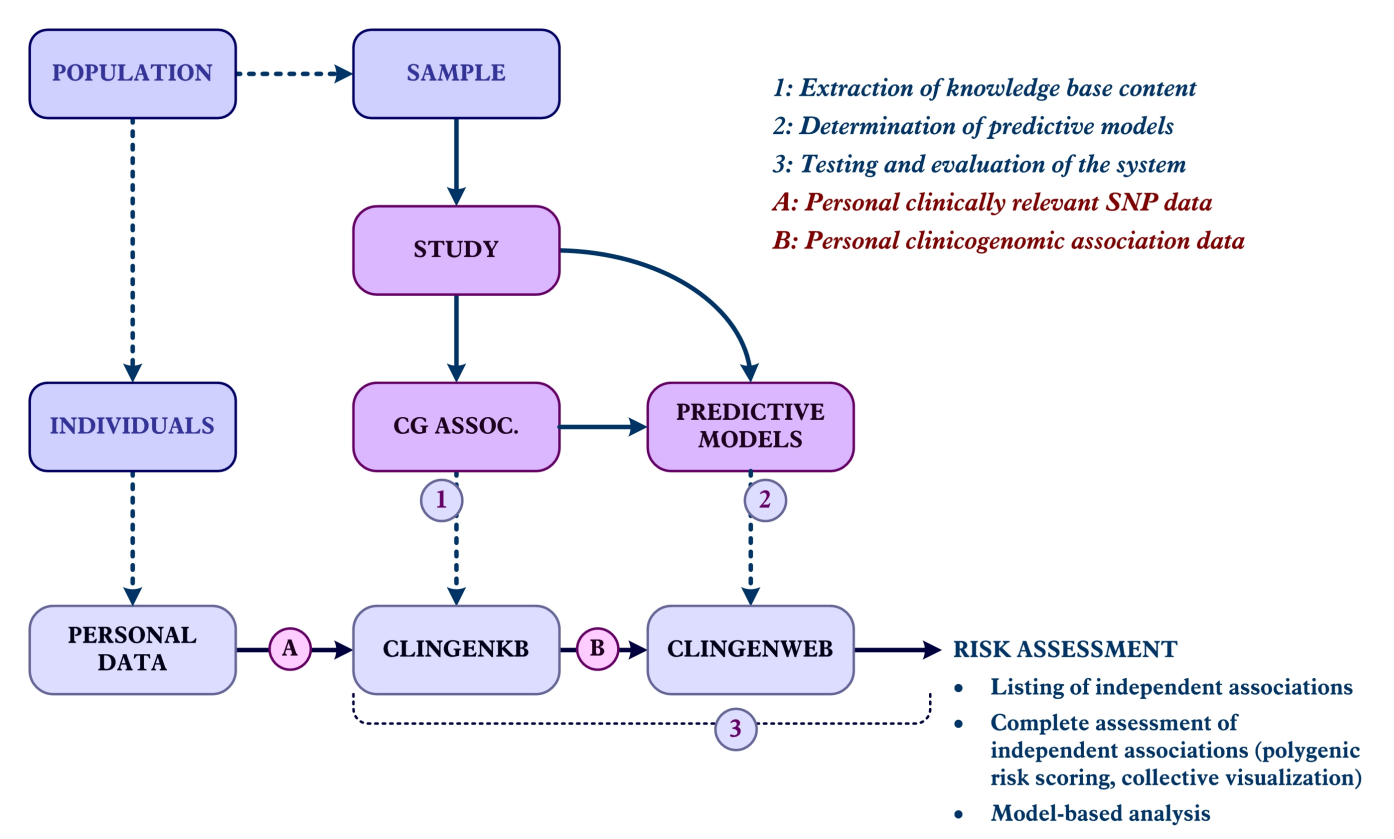
Main steps of the evaluation process. SNP=single nucletotide polymorphism; CG-ASSOC.=clinicogenomic association; CLINGENKB=clinicogenomic knowledge base; and CLINGENWEB=clinicogenomic web application.

Figure 1 shows the main workflow of the process. First, the medical literature and knowledge sources to extract clinicogenomic associations between SNP alleles and increased prostate cancer risk are investigated. Additionally, the published predictive genomic models assessing individual prostate cancer risk are searched. In parallel, to evaluate our system with real data, we have gathered the personal SNP data (23andMe files) of individuals with prostate cancer and control samples. These data files are used in the evaluation phase to infer personal clinicogenomic associations based on ClinGenKB in the final stage. The independent associations and model-based prostate cancer risk assessment approaches are evaluated and compared using real personal clinicogenomic data and external data, for example, body mass index (BMI).

### Extraction of the Independent Clinicogenomic Associations

#### Collection of Associations

Since the completion of Human Genome Project, SNP-disease relationships have been extensively researched and published in the medical literature. Results of these studies are mostly collected in structured and/or narrative forms, from several clinicogenomic knowledge sources. To develop a clinicogenomic knowledge base for prostate cancer risk, we determined reliable medical sources and collected clinicogenomic associations in a standardized form.

In our study, to extract these associations, we have preferred to utilize the publicly available knowledge sources, for example, genome-wide association studies (GWAS) catalog, SNPedia, and Cancer GAMAdb. We have selected the clinicogenomic associations between SNPs and increased prostate cancer risk from these knowledge sources, excluding studies about gene-environment (eg, nutrition, drugs, chemical agents, etc) interactions. In addition, we have ignored clinicogenomic associations measuring SNP effects on the aggressivity and mortality of the prostate cancer.

As the SNP nomenclatures and notations are represented heterogeneously among different medical sources, the correct unification and standardization of identifiers had to be the initial step. We have checked all of the selected associations and matched their reference single nucleotide polymorphism identifiers (rsIDs) and alleles using Single Nucleotide Polymorphism database (dbSNP). The SNP rsIDs, which had been merged with another SNP, were updated, and allele values, which had been identified based on reverse strand, were transformed to the forward strand.

#### Selection of Suitable Associations

Generally, there is more than one odds ratio (OR) for every SNP-disease association in various GWAS data warehouses, depending on the diversity of studies. A selection strategy is proposed to solve these value redundancies and conflictions. For the clinicogenomic association set, we have developed a four-phased selection approach to determine a reasonable value per SNP allele.

In the first phase, because all test data was gathered from Caucasians, we have obtained the clinicogenomic association values from studies, which were performed on this race group. If there weren’t any studies in the Caucasian populations, we would’ve preferred to use results from the mixed population as a second choice, and results from other races (Africans, Asians, etc) as the last choice. In the second phase, we have assessed the study type, for example, meta-analysis or research study, and preferred meta-analysis results. After that, if we still had more than one association value, we calculated the citation number of referenced articles. Finally, we have selected the highest OR, when needed (Table 1). With this approach, we extracted one OR value for every single SNP from the knowledge sources.

**Table 1:** Selection criteria for extracted associations.

Phase	Category	Order of preference
1	Race and ethnicity	Caucasians; mixed; other races (Africans, Asians, etc)
2	Study type	Meta-analysis; research study
3	Credibility of journal	Highest number of citations
4	Odds ratio	Higher number

#### Evidence Degree Assignment to Clinicogenomic Associations

There are still many biases and errors in the interpretation of genetic association studies. Ideally, we would prefer to evaluate association values to sort out all sources with a bias (study design, genotyping problems, publication bias, etc) of studies, but it becomes infeasible due to the time and effort needed by the professional domain experts. So, a degree of evidence quality is developed to rank all association values that are assigned.

During the extraction of clinicogenomic associations for prostate cancer, we have generated a simple approach using some indirect metrics to determine the quality of evidence degree for every association to assess the clinical utility. There are three major criteria that are used to determine the dimensions of evidence; credibility of referenced article, reliability of the study, and the scientific familiarity of SNP-disease relationships. To calculate the credibility of the referenced article, we have used the citation number of the article, the type of study, and the number of authors. Then the reliability of the study is determined based on the sample size (number of cases and controls), race, and ethnicity status are also considered. To evaluate the scientific familiarity of SNP-clinical condition relationship, we have calculated the number of the scientific articles about the SNP-prostate cancer relationship in PubMed, and the number of cumulative models, which involves the SNP allele under evaluation. These criteria are summarized in Table 2. Finally, the degree of evidence quality was calculated as the arithmetic average of all parameters for each association.

There are many SNPs reported with minor association degrees to predict prostate cancer risk. For a physician, it is impossible to interpret all disease relevant SNPs to determine the appropriate clinical action. Thus, to present an overview of the personal risk SNPs as a whole, we have categorized the magnitude of impact and the evidence degree values of associations into three classes as strong, moderate, and weak. The thresholds for the magnitude of impact (OR) were determined as strong (≥2.50), moderate (≥2.00 and <2.50), and weak (<2.00) (Table 2).

**Figure 2:**
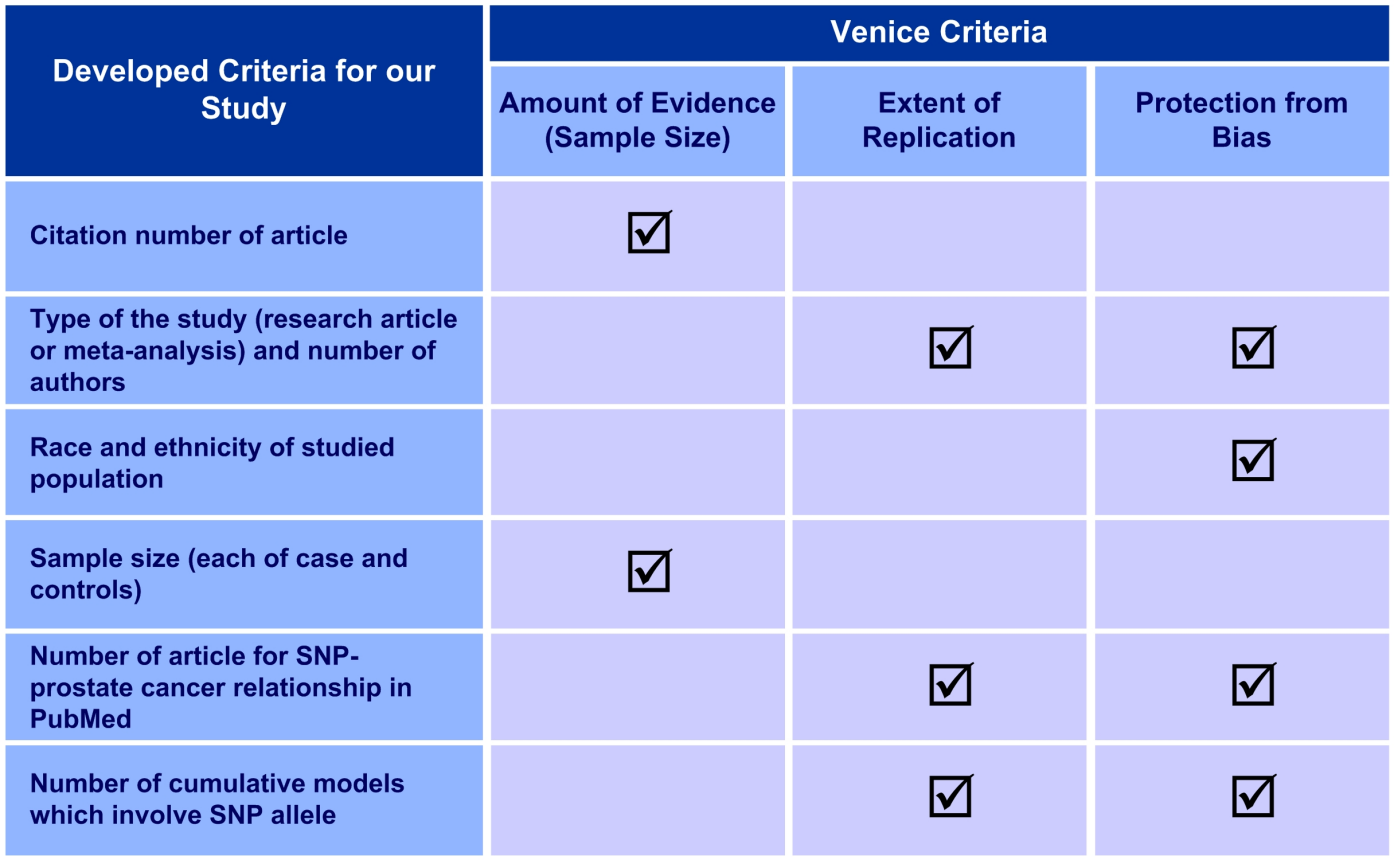
Matching of our parameters and Venice criteria. SNP=single nucleotide polymorphism.

We have also extracted indirect metrics corresponding to Venice criteria to assign an evidence degree using PubMed publications and our knowledge sources (Figure 2 shows this image). This method has a potential for an automated evidence value assignment, but needs to be validated in a separate study.

**Table 2:** Evidence degree assignment criteria for clinicogenomic associations.

Order of preference		Value
**Credibility of referenced article**		
	Citation number of article	(1-15)=1, (16-50)=2, and (>50)=3
	Type of study and number of authors	(Research article and author number <10) =1; (research article and ≤10 author number <35)=2; (research article and ≤35 author number)=3; (meta-analysis and author number<7) =2; and (meta-analysis and ≥7 author number)=3
**Reliability of study**		
	Race and ethnicity of studies population	Other races (Africans, Asians, etc)=1; mixed=2; and Caucasians=3
	Sample size (each of case and controls)	(<100)=1; (≥100 and <1000)=2; and (>1000)=3
**Scientific familiarity of SNP-disease relationship**		
	Number of article for SNP-prostate cancer relationship in PubMed	(<7)=1; (≥7 and <19)=2; and (≥20)=3
	Number of cumulative models which involve SNP allele	None=1, (<3)=2, and (≥3) =3
**Degree of evidence quality**		
		=Total value/6 (<1.5)= weak; (≤1.5 and <2.3)= moderate; and (≤2.3)= strong

### Risk Assessment and Reporting Approaches

As explained in the first part of this miniseries, there are different types of risk assessment and reporting approaches, for example, listing of clinicogenomic associations and their effects as independent associations, complete representation of these SNPs using visualization techniques, calculation of disease risk using polygenic risk scoring and model-based approaches, etc. In this study, we have focused on the different models for prostate cancer.

Because most of the genomic associations have small degrees of impact, cumulative models, which contain a few critical SNPs, have been proposed previously to predict the disease risk. We have extracted cumulative models for prostate cancer risk assessment through PubMed searches.

The rsIDs and allele values of SNPs contained in models were checked and adapted to forward genomic strands based on dbSNP entries. Models, which involve additional external parameters, such as family health history, are also collected. Finally, the reference tables for all models containing the total impact of involved parameters and corresponding risk values are generated.

Among risk assessment tools other than cumulative models, there are ongoing efforts utilizing different data mining algorithms to interpret GWAS data for building various predictive models. In order to present how these modeling approaches could be implemented in our prototype system, we also included two such examples into our study. These probabilistic models are based on the works of Yücebaş and Aydın Son to assess prostate cancer risk, and were developed through a hybrid approach combining support vector machine (SVM) and Iterative Dichotomiser 3 (ID3) decision tree (DT) based on “A Multiethnic Genome-wide Scan of Prostate Cancer” dataset from the database of Genotypes and Phenotypes (dbGaP) (study accession no., phs000306, and version 2) [6,7]. The first hybrid model (only SNP model) includes 33 SNPs and their alleles, and the accuracy, precision, and recall values of this model are 71.6%, 72.69%, and 68.96%, respectively [6,7]. The second hybrid model originally was developed by integrating genotyping and phenotyping data, and contains 28 SNPs, along with clinical features; BMI, alcohol intake, and cigarette smoking. The accuracy, precision, and recall values of this model for the integrated model are 93.81%, 96.55%, and 90.92%, respectively [6,7].

Similar to cumulative models, to prepare these hybrid models, first, we checked rsIDs and adapted allele values of contained SNPs to forward deoxyribonucleic acid strands using dbSNP. After that, we have converted the results of hybrid models as association sets. Finally, we have prepared a reference table for both of the genomic risk models containing SNP parameters.

Polygenic risk scoring is an extension of cumulative model-based approaches. Different types of polygenic prediction models were developed to combine the impact of disease associated SNP data, for example, count method, log-odds method, multiplicative model, etc. The count method is the calculation of the total count of independent genomic risk alleles. The log-odds method adds together the natural logarithm of the allelic OR for each risk allele [8]. DTC testing companies typically employ a multiplicative model to calculate life time risk in the absence of an established method for combining SNP risk estimates, that is multiplication of OR of each genotype and average population risk [9].

### Preparation of Test Data

To evaluate the ClinGenKB and the ClinGenWeb platforms as a part of our use case scenario, we have gathered real data (23andMe files) from the Personal Genome Project [10]. In this publicly available resource, genomic, environmental, and human trait data are integrated together. There were four 23andMe files that belonged to men who have been diagnosed with prostate cancer. All of these patients were Caucasian men, over 60 years of age. To build a demographically matched control set, we have selected all the Caucasian men older than 60 years of age as control samples. Through the Personal Genomes Project’s website, we have acquired 23andMe files of 15 individual healthy Caucasian men over the age of 60 (Table 3).

Before the evaluation of the proposed workflow and the framework, first, a personal clinically relevant SNP (CR-SNP) data file for prostate cancer patients from their original 23andMe files are generated based on the clinicogenomic associations. Then the clinicogenomic associations and these test data are transferred into the ClinGenKB. Personal clinicogenomic associations were acquired by processing the personal CR-SNP data with a smart query based on the ClinGenKB. After that, acquired clinicogenomic associations were transferred into the ClinGenWeb. Also, some relevant personal health data were transferred from the Personal Genome Project website to the ClinGenWeb to be used in the interpretation of disease risks based on the models. Finally, the validity of implemented models and approaches are compared and discussed.

**Table 3:** Characteristics of genomic data owners.

Participant	Prostate cancer	Ancestral origin	Birth year
01-hu1213DA	Yes	Germany-Norway	1937
03-huD889CC	Yes	Ireland	1938
07-hu28F39C	Yes	United States	1943
13-hu6ED94A	Yes	United States-Austria	1950
02-hu59141C	No	United States-Canada	1937
04-huF7E042	No	United States-United Kingdom	1939
05-hu75BE2C	No	United States	1939
06-hu56B3B6	No	United States	1941
08-huB59C05	No	United States-Ireland	1943
10-hu7A2F1D	No	United States-Germany	1947
12-huD57BBF	No	United States	1949
14-huD7960A	No	Hungary-Ukraine-Russia	1951
15-hu2E413D	No	United States	1952
16-hu76CAA5	No	United States	1952
17-huA720D3	No	United States-United Kingdom	1953
18-hu63DA55	No	United States	1953
19-hu43860C	No	United Kingdom-Hungary	1954
20-huD00199	No	Germany-Poland	1954
21-huAC827A	No	United States-Sweden	1954

### Evaluation of Test Data

In prostate cancer, known relevant SNPs mostly have a modest OR. Therefore, in the evaluation phase, we have assessed the total impact of the independent relevant associations based on four approaches, that is the number of SNPs based on the dominant model, the number of SNPs based on the additive model, the evidence-impact-SNP degree based on the dominant model, and the evidence-impact-SNP degree based on the additive model. The “number of SNPs”, are calculated as the total count of existing relevant SNPs. In the dominant type of this model, only the count of relevant SNPs is considered, but in the additive type, the impact of homozygote SNPs is weighted twice as much compared to heterozygote SNPs. In the evidence-impact-SNP approach, for every existing SNP, we have calculated an impact degree using the evidence degrees (1, 2, and 3) and the impact degrees (1, 2, and 3). Also, similar to the number of SNPs calculated, for the additive type we have assigned 1 and 2 to heterozygote and homozygote SNPs as weighting coefficients, respectively.

After that, all the cases and controls are evaluated based on the predictive cumulative and probabilistic models. Then, results for all of the cases and controls were interpreted and compared. In the second hybrid model, where associations are based on both genotyping and clinical data, SNPs and BMI, smoking and alcohol consumption data are used. Here, due to a lack of the clinical data, the risk for some individuals could not be assessed.

In addition to the genetic factors, there are various comorbidities, sociodemographic characteristics, and environmental and behavioral exposures that are proposed as confounders of the prostate cancer (Table 4). Therefore, we have analyzed the personal, clinical, and the environmental characteristics, which are meaningful for the prostate cancer pathogenesis, as the last step of our evaluation.

**Table 4:** Example list of several risk and protective factors for the prostate cancer [2,3].

Risk category	Parameters
Sociodemographic data	Age, family health history, ethnicity, and race.
Environmental sources	Nutrition and diet (animal fat, fruits, legumes, yellow-orange and cruciferous vegetables, soy foods, dairy products, fatty fish, alcohol, coffee, green tea, modified citrus pectin, and pomegranate).
	Supplements (multivitamins, vitamin E -with or without selenium, folic acid, zinc, calcium, vitamin D, retinoid, and zyflamend).
	Drugs (5 alpha-reductase inhibitors, nonsteroidal antiinflammatory drugs, statins, and toremifene).
	Medical procedures (vasectomy, barium enema, hip or pelvis x-rays, and external beam radiation therapy for rectal cancer).
	Tobacco use (tobacco products, smoking).
Personal health status (internal environment)	Medical conditions (prostatitis, prostatic intraepithelial neoplasia, syphilis, skin basal cell carcinoma, and benign prostate hyperplasia).
	Anatomic measurements (high body mass index).

## Results

### Independent Associations for Prostate Cancer

Initially, we have determined 87 SNP alleles from the GWAS catalog, 32 SNP alleles from the SNPedia, and 236 SNP alleles from the Cancer GAMAdb, which are all associated with increased prostate cancer risk. Through the extraction and selection processes of SNP-prostate cancer risk associations, we have excluded redundant, conflicting, and incomplete associations. Finally, a total of 209 independent associations for increased risk of prostate cancer from the studied knowledge sources were acquired. Next, the evidence and the impact categories are assigned to these associations (see Multimedia Appendix 1). The overall assessment of all these different types of clinicogenomic associations is summarized in Table 5.

**Table 5:** Distribution of clinicogenomic associations.

	Evidence degree			
Impact degree	Strong	Moderate	Weak	Total
Strong	0	5	2	7
Moderate	0	3	1	4
Weak	42	123	33	198
Total	42	131	36	209

### Cumulative Models for Prostate Cancer

Cumulative models are the combination of the impact of several clinicogenomic associations using arithmetic operators. For some SNPs, only homozygote alleles are involved in the models (recessive model), but mostly heterozygote SNPs (dominant model) are part of the cumulative models. Both in dominant and recessive models, the values of risk SNPs are accepted as one unit of impact. Alterations of SNPs’ impact values regarding homozygote and heterozygote alleles are defined as an additive model. The dominant and recessive models as examples of the cumulative predictive models retrieved from the scientific literature, and the SNP alleles included in each of the cumulative models are listed in Table 6.

In addition to Table 6, three of these cumulative models (17-SNP_Helfand, 5-SNP_Zheng and 5-SNP_Salinas) were enhanced using family health history as an additional parameter and combined SNP-family health history models were produced [11-13].

In the cumulative models, the existence of each association contributes to the total score. For example, in the 5-SNP_Zheng model, there are five different SNPs. The genetic model is dominant for three SNPs (rs1447295-A, rs16901979-A, and rs6983267-G) and recessive for the others (rs1859962-G, rs4430796-A). For dominant models, homozygote and heterozygote combinations of alleles are identified as a risk factor in the same degree. For recessive models, only homozygote combinations are considered as risk factors, whereas heterozygote combinations are accepted as harmless. Through analysis of a patient’s genotype, the total impact values of clinicogenomic associations are determined and calculated additively. Besides the SNP associations, the existence of prostate cancer in family health history can be included as an additional impact factor.

The reference table for 5-SNP_Zheng model is presented as an example in Table 7. If patients without family health history have only one impact factor, the risk of having prostate cancer increases by 1.5, compared to those who have none of the impact factors. If a patient has all five risk SNPs with specified alleles, and a positive family health history for prostate cancer, the total impact is calculated to be 6. According to Table 7, this would correspond to an increased risk of 9.46 for having prostate cancer when compared to the general population. Full reference tables for all cumulative models are provided in the Multimedia Appendix 2 [11-16].

**Table 6:** Examples of cumulative risk prediction models for prostate cancer.

	17-SNP_Helfand [11]	9-SNP_Helfand [14]	5-SNP_Zheng [12]	5-SNP_Salinas [13]	4-SNP_Nam [15]	3-SNP_Beuten [16]
rsIDs and risk allele						
rs1819698-T						Dominant
rs2710646-A		Recessive				
rs721048-A	Recessive					
rs10934853-A	Dominant					
rs2736098-A	Recessive					
rs401681-C	Dominant					
rs1800629-A					Dominant	
rs2348763-A					Recessive	
rs1447295-A	Dominant	Dominant	Dominant	Dominant	Dominant	
rs16901979-A	Dominant	Dominant	Dominant			
rs16902094-G	Dominant					
rs445114-T	Dominant					
rs6983267-G	Dominant	Dominant	Dominant	Dominant		
rs6983561-C				Dominant		
rs10993994-T	Recessive	Recessive				
rs10896450-G	Dominant	Dominant				
rs11228565-A	Dominant					
rs12439137-G						Dominant
rs2470152-T						Dominant
rs11649743-G	Recessive					
rs1859962-G	Recessive	Recessive	Recessive	Recessive	Recessive	
rs4430796-A	Dominant	Dominant	Recessive	Recessive		
rs8102476-C	Dominant					
rs5945572-A	Dominant	Dominant				

**Table 7:** Reference table for 5-SNP_Zheng model.

Total impact	Odds ratio (95% CI), without FHH^a^	Odds ratio (95% CI), with FHH^a^
0	1.00 (by definition)	1.00 (by definition)
1	1.50 (1.18-1.92)	1.62 (1.27-2.08)
2	1.96 (1.54-2.49)	2.07 (1.62-2.64)
3	2.21 (1.70-2.89)	2.71 (2.08-3.53)
4	4.47 (2.93-6.80)	4.76 (3.31-6.84)
5	4.47 (2.93-6.80)	9.46 (3.62-24.72)
6	-	9.46 (3.62-24.72)

^a^ FHH = family health history

### Probabilistic Models for Prostate Cancer

In this study, we used two types of probabilistic models from Yücebaş and Aydın Son based on a hybrid (SVM+ID3 DT) approach; namely, first (only SNP) and second (SNP-Environmental Combined) [6,7]. When the first hybrid model (only SNP model) from Yücebaş and Aydın Son is interpreted, we have captured 154 different association sets containing the combination of several different SNPs and alleles [6]. In the second genotype-phenotype integrated model, we acquired 23 association sets containing 28 SNPs and their alleles along with BMI, smoking, and alcohol usage [7]. The complete associations of the hybrid models are listed in Multimedia Appendices 3 and 4.

In these probabilistic models, if an individual accounted for all parameters on one branch (ie, an association set), this individual has a prostate cancer risk with the accuracy, precision, and recall values of total model as presented in references [6,7]. Table 8 presents an example of the reference table for association sets of the genotype-only hybrid model.

**Table 8:** Reference table for the probabilistic only SNP model.

Branch_id	Total count of SNPs
Branch_ 1	4
Branch_ 2	4
Branch_ 3	7
….	….
Branch_ 154	2

### Evaluation Results for Test Data

#### Overview

In the evaluation phase, we have studied four cases and 15 controls, which consisted of Caucasian men, age 60 years or older, and regarding independent clinicogenomic associations and risk prediction models.

Complete results of test and evaluation processes (independent association assessment, model-based evaluation, and clinical and environmental evaluation) are provided in Multimedia Appendix 5.

#### Results for Independent Associations

In prostate cancer, known relevant SNPs mostly have a modest OR. Therefore, in the evaluation phase, we have assessed the total impact of the independent relevant associations based on four approaches, that is the number of SNPs based on the dominant model, the number of SNPs based on the additive model, the evidence-impact-SNP degree based on the dominant model, and the evidence-impact-SNP degree based on the additive model.

The comparative evaluation results of individual clinically relevant SNPs of case and control groups regarding categorical distribution of evidence quality and impact degrees are in Multimedia Appendix 6. In these approaches, case groups were divided into two or three different subsets (three patients with high values and one patient with a low value for the dominant models, and two patients with high, one patient with moderate, and one patient with low values in terms of additive models). In control groups, there were, in particular, five people (21-huAC827A, 15-hu2E413D, 08-huB59C05, 17-huA720D3, and 06-hu56B3B6) with values higher than all cases observed. However, it must be remembered that, in the complete assessment of all SNPs, due to the remarkable number of relevant SNPs that were not analyzed, the results might be distorted.

#### Results for Cumulative Models

Due to a lack of family health history data of individuals, we couldn’t use this data to calculate cumulative risks. In our limited number of cases, cumulative models did not have meaningful results. But, similar to the complete evaluation of independent associations, it must be considered that, nonanalyzed SNPs could be distorting the results. Results of these cumulative models are summarized in Table 9.

**Table 9:** Summarized results for cumulative models.

	Case			Control		
	Odds ratio≥2.5	Odds ratio<2.5	Unknown	Odds ratio≥2.5	Odds ratio<2.5	Unknown
17-SNP_Helfand	1	-	3	2^a^	10	3
9-SNP_Helfand	1	3^b^	-	1^c^	12	2
5-SNP_Zheng	-	4	-	-	15	-
5-SNP_Salinas	-	4	-	-	15	-
4-SNP_Nam	-	4	-	-	15	-
3-SNP_Beuten	-	2	2	-	13	2

^a^ 02-hu59141C, 12-huD57BBF

^b^ 01-hu1213DA, 03-huD889CC, and 07-hu28F39C

^c^ 17-huA720D39

#### Results for Probabilistic Models

Regarding the probabilistic model-based interpretations; the only SNP model from Yücebaş and Aydın Son [6] wasn’t successful in terms of predicting the cases. In the second model [7], where genotype and phenotype data were integrated, one patient was determined as being under risk, two patients couldn’t be evaluated because of data incompleteness (smoking and alcohol consumption data), and one patient (03-huD889CC) was determined as being risk free. In control samples, only one individual (04-huF7E042) was determined as being in a risk group, but six individuals were determined as being risk free. There were eight individuals of this group that couldn’t be evaluated due to data incompleteness. Although this model was produced for those of African American descent, and even though we had a limited number of cases and controls for the evaluation process, it was still the most successful approach when compared to the others. Interestingly, a patient (03-huD889CC) was determined as the risk free, and this patient was also determined as being in a low risk group according to complete assessment approaches.

#### Clinical and Envirobehavioral Evaluation

Prostate cancer is a polygenic multifactorial disease, and both environmental and genetic factors take important roles in its pathogenic mechanism. Therefore, if we analyze the genomic risks with clinical and environmental characteristics, we can infer more accurate results. Characteristics of cases and controls regarding clinical and environmental risk factors for prostate cancer are summarized in Table 10.

In envirobehavioral and clinical evaluation, it was found that patient “03-huD889CC” had previously been diagnosed with syphilis. In prior publications, syphilis has been reported as a risk factor for prostate cancer [2]. The healthy individuals, who had a higher risk than controls, namely “06-hu56B3B6”, had basal cell carcinoma, and “21-huAC827A” had hypogonadism, that is a low level of testosterone. And both of these clinical conditions are known to decrease the prostate cancer risk [1,2]. Also, “06-hu56B3B6” and “17-huA720D3” used several risky, protective drugs and supplements regarding prostate cancer risk. In patients “08-huB59C05” and “15-hu2E413D”, we did not have enough data to evaluate the risk and protective factors. In the health records of some cases and controls, there was some data about nutritional status, physical activity, and usages of supplements data, etc. But, all this data wasn’t useful during the evaluation due to a lack of precise measurement information (eg, amount, period, duration, etc).

**Table 10:** Clinical and environmental risk factors of cases and control.

Group	Individuals	Risk factors	Protective factors
Case	01-hu1213DA	Hypercholesterolemia, BPH^a^	
Case	03-huD889CC	Syphilis	
Case	07-hu28F39C	Hypercholesterolemia, BPH^a^, and lipitor	
Case	13-hu6ED94A	Obesity, hypercholesterolemia, and simvastatin	
Control	02-hu59141C	Obesity, multivitamins	T2DM^b^, vegetable servings, and regular physical activity
Control	04-huF7E042	BPH^a^	TURP^c^
Control	05-hu75BE2C		Regular physical activity
Control	06-hu56B3B6	Obesity, hypercholesterolemia, chlamydia infection, alcoholism, ibuprofen, multivitamin, folic acid, vitamin E, and selenium	Basal cell skin cancer, lycopene, and pomegranate
Control	08-huB59C05	Obesity	
Control	10-hu7A2F1D	Hypercholesterolemia, atorvastatin	Nonmelanoma skin cancer, regular physical activity
Control	12-huD57BBF	Hypercholesterolemia, BPH^a^ Simvastatin, aspirin, and vasectomy	Regular physical activity
Control	14-huD7960A	Overweight, hypercholesterolemia, and BPH^a^	T2DM^b^
Control	15-hu2E413D	Overweight	
Control	16-hu76CAA5	Overweight, aspirin	Omega-3 fish oil
Control	17-huA720D3	Hypercholesterolemia, aspirin, and multivitamin	Phytosterols, omega-3 fish oil, and melatonin
Control	18-hu63DA55		Omega-3 fish oil
Control	19-hu43860C	Overweight, hypercholesterolemia, and lovastatin	Nonmelanoma skin cancer
Control	20-huD00199	Overweight, hypercholesterolemia, and atorvastatin	
Control	21-huAC827A	Overweight, hypercholesterolemia, and simvastatin	Hypogonadism

^a^ BPH = benign prostate hyperplasia

^b^ T2DM = type II diabetes mellitus

^c^ TURP = transurethral resection of the prostate

## Discussion

### Principal Results

In this study, we have extended the current architecture of a centralized national EHR, NHIS-T, and developed two complementary capabilities, a knowledge base (ClinGenKB) and a reporting application (ClinGenWeb), to predict the risk of diseases using SNP data.

With respect to interoperability, Health Level 7 Clinicogenomic Work Group (HL7 CG-WG) develops several standards and guidelines, and tries to overcome the chasm between the genomic laboratory and the clinical practice. In comparing current and required infrastructure characteristics, and determining a few terminology standards for genome enabled messaging, we reason NHIS-T can be adapted to HL7 CG-WG.

The unique identification of SNP data is a critical issue in clinical genomics. In our system, due to simplicity and easiness, we proposed to use rsIDs and allele values for identification of SNPs. But, to avoid any inconsistencies, it is crucial to remember that, some rsIDs have been merged over time. For this reason, SNP numbers must be checked out based on the dbSNP, and transformed into current values if required. Additionally, as different genomic strand types are the preferred choice among some clinicogenomic knowledge sources and publications, the standardization of strand identification is another important point for SNP data incorporated into clinical systems.

Regarding clinical terminology, we prefer to use existing NHIS-T standards, for example, International Classification of Diseases and Related Problems, Tenth Revision (ICD-10) for disease identification. For new data types (model name, model type, etc), we produced our own specific value categories.

To store and process the huge amount of raw variant files, in our architecture, we have proposed to store the raw and/or processed genomic data in the genomic laboratory databases, and only to share clinically relevant variant data and/or clinicogenomic association information between partners. To derive CR-SNP data from personal SNP data, we need to use a CR-SNP resource. This resource was designed as part of a national level clinicogenomic knowledge base. This knowledge base is also utilized to transform CR-SNP data to clinicogenomic associations.

As it is emphasized in the literature, one of the most critical components of the genome enabled EHRs is the development of a national level knowledge base for clinicogenomic information. This capability must be kept up to date and manually curated by domain experts. In our study, we have developed a prototype knowledge base (ClinGenKB), which includes clinicogenomic associations for prostate cancer risk prediction.

Several different approaches are proposed to define clinical impact and evidence qualities of clinicogenomic associations in various knowledge sources. But there is still a lack of structured, objective, and comprehensive methodologies for matching, selecting, and merging different studies. In our prototype, we have proposed a simple methodology, but the best methods of determining standards to calculate, limit biases, and limit faults still need to be investigated in future clinicogenomic association studies.

ClinGenWeb is a prototype for the end-user systems that provides interpretations of the clinicogenomic associations. To evaluate our system, we have used real data from the Personal Genome Project. Collected data included 23andMe data files, ages, ethnicities, ancestral origins, clinical data, and some behavioral parameters. Age and ethnicity are extensively accepted as proven risk factors for prostate cancer. All of our cases and controls were selected from Caucasian men over 60 years old. The risk for prostate cancer is 2 in 16 for men 60 through 69 years old, and 1 in 9 for men 70 years and older [17].

ClinGenWeb uses both complete and model-based interpretations for clinicogenomic associations. Independent associations may have very little importance for clinical processes alone, but in complete interpretation, we tried to interpret all relevant data as a whole. After analyzing our results, we concluded that cases and controls could be divided into two or three different risk groups as a result of genetic heterogeneity. With the commissioning of whole genome sequencing/whole exome sequencing (WGS/WES) in clinical practice, similarity measurements of clinically relevant SNP patterns may be a new way of producing predictive models in genomic medicine, but this approach needs to be supported with more phenotypic data, and needs to be tested in larger study samples.

There are several cumulative models proposed to predict prostate cancer, but we couldn’t acquire meaningful results with these models in our subjects. Another original approach was to use the probabilistic (SVM+ID3 DT) model-based associations. However, the only SNP model of this approach was not successful, but the second model, which integrates genotype and clinical data, was partly consistent. Unfortunately, the number of available holistic envirogenomic models that could be implemented here is limited. The probabilistic model utilized was produced for men of African American, Latin, and Japanese descent, and we have used the submodel template generated for African American individuals, as their genetic background is expected to carry a higher number of common SNPs with the Caucasian population than Latin or Japanese populations.

Another critical point is that clinical, environmental, and behavioral data can be used to explain pathogenic and clinical heterogeneity, and to clarify the complexity of results. With the support of clinical and behavioral data, we could interpret some contradictory results. Because, most of the environmental and behavioral data wasn’t stored in EMR/EHRs in a structured manner, we generated the functionality to add these types of data at the end-user level.

Due both to the bipartite structure of our interpretations (ie, conversion of CR-SNP into clinicogenomic associations and final clinical interpretations of associations), and the fact that the final interpretation was accomplished at the end-user side, we combined both clinicogenomic associations and external parameters (such as BMI), which have been recorded or tracked by end-users to support the decision making.

### Limitations

Complete implementation of SNP data incorporated NHIS-T in real systems was not possible due to the regulative and the technical issues at this stage. So, we restricted our focus to develop complementary capabilities as prototypes for NHIS-T, namely, the ClinGenKB and the ClinGenWeb, which specifically targeted prostate cancer risk prediction.

GWAS research is based on the “common disease, common variant” hypothesis. However, some authors proposed that common variants can explain only a modest part of complex diseases and so the “common disease, rare variant(s)” hypothesis was recently put forward [18]. Clinicogenomic associations used to build the knowledge base in this study are based on recent developments in the GWAS research and literature. In our study, we have only used SNP data, but recent studies show that different variants (Copy Number Variations, etc) are also responsible for clinical conditions.

Also in the ClinGenKB, our critical focus was to generate a structured clinicogenomic representation for only risk prediction for prostate cancer. But, in the literature, there are several kinds of information related to different stages of clinical decision processes, for example, prognosis, pharmacogenomic, etc. In the real world project, this prototype has to be enhanced with additional types of associations and diseases.

We obtained case and control data from the Personal Genome Project to evaluate our system, but the number of cases and controls were so limited. To determine the value of this system in clinical settings, more comprehensive data on genomic, environmental, family health, and clinical conditions are needed. Unfortunately, none of the cases and controls had family health history data, and we couldn’t involve this critical parameter in our evaluation processes. Existing clinical data about subjects didn’t reflect the clinical and pathological heterogeneity of the prostate cancer. In particular, we did not have precise measurement information (amount, period, duration, etc) about behavioral characteristics of subjects (diet, physical activity, supplements, etc), and we couldn’t interpret the possible effects of these parameters on prostate cancer risk.

Another limitation was in aligning the terminologies of the clinical and bioinformatical domains in a consistent way. ICD classification is accepted as a standard for disease classification in many countries including Turkey. But ICD-10 is not useful to manage all levels of clinical, pathologic, and genetic heterogeneities. It is expected that it will be managed in the next version, ICD-11 that will be released in 2015 and the new release can be integrated with other medical terminologies such as Systematized Nomenclature of Medicine Clinical Term (SNOMED-CT) [19]. Nevertheless, as proposed earlier, it is an unavoidable requirement to develop a new taxonomy of diseases, which will be based on information commons and a knowledge network, combining molecular data, social data, environmental data, clinical data, and health outcomes [20].

In the current study, due to the ethnic characteristics of our subjects, we have primarily preferred the studies performed with Caucasians to collect the clinicogenomic associations from the literature. But, the terms of ethnicity and race are sociocultural constructs affected by both biological and environmental factors. For this reason, for a real world NHIS-T system, genotyping data from the Turkish population is needed to build the working knowledge base.

Also, predictive models that will be used in clinical settings need to be validated. Especially, we need approaches to assess the complete analysis of clinically relevant SNPs. With the commissioning of WGS/WES in the clinical practice, similarity measurements of clinically relevant SNP patterns may be a new way to produce predictive models in genomic medicine, but this approach needs to be enhanced with further phenotypic data, and to be tested in large study samples.

On the other hand, the number of available holistic envirogenomic models is limited. As most of the complex diseases are progressing as an interaction of genomic and environmental factors, more envirogenomic data also need to be developed to build predictive disease models.

### Comparison With Prior Work

GeneInsight Suite is an impressive application environment to evaluate and share sequencing based test results. GeneInsight Clinic can be integrated with EMRs or can be used as a standalone system. It manages knowledge, and facilitates reporting. GeneInsight Network (VariantWire) provides the mechanism to connect laboratories and providers. Interpretations of sequencing based tests are shared with corresponding caregiver organizations using this system. GeneInsight Suite allows clinicians to receive updates when new information on previously unknown variants is certified for clinical use.

There are critical differences between the proposed system and GeneInsight. First, our system is designed as part of a central national level EHR. In the United States, the architecture of EHR systems is more federated. Both systems include a knowledge base and applications for the end-users.

In GeneInsight, the interpretation and reinterpretation of critical variants are reported for clinical use. These interpretations do not involve external data, which is not included in the EMR. But, in the proposed system, the clinical interpretation of SNP data is divided into two sequential processes, that is the conversion of CR-SNP into clinicogenomic associations, and the clinical interpretation of them. Final interpretation is completed at the end-user application, and so it is possible to use additional data for the risk prediction (environmental, behavioral, etc). These processes are finalized based on predictive models and automated analysis techniques.

### Conclusions

Today, the health care systems are continuously evolving and transforming under the influence of developments in technology and globalization. A revolutionary paradigm shift is changing the focus of medicine from the traditional provider-centric approach to patient-centric personalized medicine. This paradigm shift, dramatically transforms clinical processes, medical education, and research in theory and practice. The commissioning of new health services based on emerging technologies (mobile health systems, pervasive applications, environmental sensors, body area sensor networks, etc) also dramatically supports these emerging trends.

But in the light of the literature on personalized medicine, we can argue that the area of biomedical informatics has not begun to show its major effect on health care systems, and the major shifting in health care practices is expected soon via genomic technologies. When we look at the big picture, we can see the emergence of evidence-based managed health care systems with knowledge discovery capabilities driven by big data and knowledge infrastructures for sustainable, fair, and effective care services.

In this respect, we consider that the next generation of health information systems will be constructed based on tracking and monitoring all aspects of individual health status through 24/7, and implementing evidence-based recommendations to empower individuals. Today, most of the personal, behavioral, and environmental data is not a subject of EMR/EHR, or even PHR contents. Characteristics of most environmental and behavioral data require frequent measurements and (nearly) continuous tracking. And, possibly if we extend PHR content (with genomic data) toward involving environmental and behavioral factors, we can add value to disease risk assessment and prediction.

As we emphasized before, a national level manually curated and accredited knowledge base is the most important component of evidence-based decision making. Based on this knowledge base, collected risk data will gain a predictive meaning, and any new discovery in clinical sciences will be reflected for individuals by the reinterpretation of collected data. At this point, we need additional and improved analytic tools based on genomic and environmental parameters. We aim to develop a knowledge repository integrating some knowledge bases with semantic technologies, and adding some automatic evaluation techniques to make it easier to extract and manually curate existing references for the domain experts.

Regarding the challenges facing health care systems, along with the effective provision of public health services and associated financial burdens, most of the important diseases are of a complex nature. In the pathogenesis of complex diseases, the interaction of genetic and environmental factors has critical importance, and ethnicity, race, and geographic factors may play distinctive roles. Hence, it is necessary to have the appropriate clinicogenomic information about the target population. Clinical data, environmental factors, and family health history are critical components, and there is a need to study the relationships between these parameters and genomic factors. Eventually, it will be required both to conduct envirogenetic studies in order to acquire original data for population, and to enhance the NHIS-T data model for collecting these types of data.

The omics area is not only represented by genomic data, and in the near future different types of omics data will be available for the routine clinical practices, for example, transcriptomics, proteomics, metabolomics, and epigenomics. Also, systems medicine offers possibilities that will increase the effectiveness of risk prediction strategies.

In addition, we aim to enhance our system by integrating data warehouses for research. With this capability, integrated genomic and environmental datasets can also be used for clinical research. We will extract the meaningful relationship patterns via this system and, by using these patterns; we can calculate the risks of groups who have similar characteristics, for example, family members or communities.

The major aim of our system is to provide true and actionable information for patients and their family practitioners. Our system will process collected data and return evidence-based recommendations to the individuals to make them responsible for their preferences and consequences. The empowerment of individuals to participate in their health care decisions is an emerging trend in personalized medicine. At this point, we need more curated information sources and visual representation approaches intended for unprofessional individuals. Areas of representation and reporting of clinicogenomic results should focus on developing new approaches, techniques, and tools.

In the last 10 years, there has been a great effort to accomplish a transformation to a national health care system based on information technologies in Turkey. But yet, practical applications of personal genomics and its integration into health care services are in its infancy, and studies about personalized medicine are at the academic level.

Our architecture and prototype, which aim to incorporate personal SNP data into the NHIS-T, are also in their preliminary stage. However, we need additional vision, research, work, and tools to extend our EHR capabilities for the future genome enabled health care systems. We believe that our work will be a starting point for a predictive and preemptive personalized national health care system.

## Multimedia Appendix 1

Complete list of independent associations.

## Multimedia Appendix 2

Reference tables for cumulative models.

## Multimedia Appendix 3

List of first probabilistic model based associations (only SNP model).

## Multimedia Appendix 4

List of second probabilistic model based associations (SNP-environmental combined).

## Multimedia Appendix 5

Complete results of test and evaluation processes.

## Multimedia Appendix 6

Analysis of total number and values of relevant personal SNPs.

## Abbreviations

BMI: body mass index
ClinGenKB: Clinicogenomic Knowledge Base
ClinGenWeb: Clinicogenomic Web Application
CR-SNP: clinically relevant single nucleotide polymorphism
dbSNP: Single Nucleotide Polymorphism database
DT: decision tree
DTC: direct-to-consumer
EHR: electronic health record
EMR: electronic medical record
GWAS: genome-wide association studies
HL7: Health Level 7
HL7 CG-WG: Health Level 7 Clinicogenomic Work Group
ICD: International Classification of Diseases and Health Related Problems
ICD-10: International Classification of Diseases and Related Problems, Tenth Revision
ID3: Iterative Dichotomiser 3
NHIS-T: National Health Information System of Turkey
OR: odds ratio
PHR: personal health record
rsIDs: reference single nucleotide polymorphism identifiers
SNP: single nucleotide polymorphism
SVM: support vector machine
WES: whole exome sequencing
WGS: whole genome sequencing
